# PCPA: A Practical Certificateless Conditional Privacy Preserving Authentication Scheme for Vehicular Ad Hoc Networks

**DOI:** 10.3390/s18051573

**Published:** 2018-05-15

**Authors:** Yang Ming, Xiaoqin Shen

**Affiliations:** 1 School of Information Engineering, Chang’an University, Xi’an 710064, China; 2 School of Sciences, Xi’an University of Technology, Xi’an 710054, China; xqshen@xaut.edu.cn

**Keywords:** vehicular ad hoc networks, authentication, conditional privacy preserving, security, certificateless signature

## Abstract

Vehicle ad hoc networks (VANETs) is a promising network scenario for greatly improving traffic efficiency and safety, in which smart vehicles can communicate with other vehicles or roadside units. For the availability of VANETs, it is very important to deal with the security and privacy problems for VANETs. In this paper, based on certificateless cryptography and elliptic curve cryptography, we present a certificateless signature with message recovery (CLS-MR), which we believe are of independent interest. Then, a practical certificateless conditional privacy preserving authentication (PCPA) scheme is proposed by incorporating the proposed CLS-MR scheme. Furthermore, the security analysis shows that PCPA satisfies all security and privacy requirements. The evaluation results indicate that PCPA achieves low computation and communication costs because there is no need to use the bilinear pairing and map-to-point hash operations. Moreover, extensive simulations show that PCPA is feasible and achieves prominent performances in terms of message delay and message loss ratio, and thus is more suitable for the deployment and adoption of VANETs.

## 1. Introduction

With the progress in human civilization and development of industrial technology, vehicles are widely popularized in modern society, which leads to such problems as traffic congestion, accidents, vehicle emissions, etc. Therefore, wide attention has been paid to deal with the abovementioned issues in both the academia and automobile industry.

Vehicular ad hoc networks (VANETs), as a key component of intelligent transport system (ITS) and a particular mobile ad hoc networks (MANETs), is promising in improving traffic management efficiency and road traffic safety [1] . Generally, a typical VANET is mainly comprised of three types of entities, i.e., the trusted authorizers (TAs), the roadside units (RSUs) installed along the roads, and the vehicles rigged with onbroad units (OBUs). The TAs maintain the whole system and communicate with the RSUs using a secure wired communication. The RSUs alleviate the burden of the TAs by performing authentication tasks, while the vehicles (OBUs) provided the wireless communication capability, which communicate with the RSUs (Vehicle-to-Infrastructure, V2I) communication and other vehicles (Vehicle-to-Vehicle, V2V) communication. Here, IEEE 802.11 p standard is used for wireless communication based on Dedicated Short Range Communication (DSRC) protocol [2,3], in which each vehicle (OBU) broadcasts the traffic-related messages (e.g., vehicle’s speed, position, turning direction and time) periodically every 300 ms. According to the received traffic-related messages, other vehicles can alter driving routes to avoid emergent braking or traffic accidents, and the RSU will inform the traffic control center to regulate the traffic for preventing potential traffic jams. Based on the hybrid architecture of V2I and V2V communication, VANETs are conducive to enhancing traffic safety, improving traffic management and optimizing traffic efficiency.

Owing to the inherent broadcast nature of the wireless channels, the communication in VANETs is vulnerable to various attacks such as eavesdropping, replaying, tampering, modification and forgery attacks, etc. Therefore, for the widespread deployment of VANETs, the security and privacy challenges must be solved [4,5].

The authentication mechanism, which consists of identity authentication and message integrity, is the key to ensuring the security of VANETs [1,5,6]. If identity authentication is not satisfied, a malicious vehicle may impersonate as a legal vehicle to broadcast messages for obtaining illegal benefits. If message integrity is not ensured, a malicious vehicle may broadcast falsified or altered messages to seriously disrupt traffic or incur serious consequences for the surrounding vehicles without being caught. Thus, authentication has to be implemented to verify a vehicle’s identity and to differentiate trustworthy messages from received ones. The digital signature technology may be used to address this problem in VANETs, the vehicle should make a signature on messages before sending them out, and the receivers will authenticate the messages before employment.

Apart from that, privacy is also important for VANETs [7,8]. The vehicle’s privacy information like current position, license number, driver’s identity and travel route must be kept confidential for a long time. For example, the leakage of vehicle’s route information will incur the grave consequences since the information may be used for crimes or traffic accident. In general, the vehicles wouldn’t want their privacy information disclosed in broadcasting messages. Therefore, the vehicle privacy must be protected.

However, the fact is that security sometimes conflicts with privacy. Especially, the former often involves some identity information and message’s origin, while the latter requires that no entity can trace a message to its generator. Thus, conditional privacy is usually considered in VANETs. That being said, the vehicle’s privacy is usually preserved in the system. If a malicious vehicle does not perform the protocol correctly (e.g., broadcasting false messages), then its privacy is revoked, in which case a trust authority (TA) will be capable to trace or retrieve the real identity of vehicle. The conditional privacy-preserving authentication (CPPA) mechanism [9,10], which is able to achieve message authentication and conditional privacy preservation simultaneously, is fully appropriate for addressing the security and privacy issues in VANETs.

Lots of existing studies on the CPPA schemes in VANETs have been carried out in recent years. We can broadly categorize these schemes into public key infrastructure-based (PKI-based) schemes [1], identity-based (ID-based) schemes [11], and certificateless schemes [12,13,14,15].

Despite having solved the key escrow problem in ID-based schemes and the public key certification management problem in PKI-based schemes, the certificateless schemes are still unsuitable for the VANETs. The reason is that such schemes [12,13,14,15] have poor performances due to the requirements of map-to-point hash and bilinear pairing operations. Compared to other cryptographic operations, these two operations are complex and time-consuming. Therefore, it is important to design a practical certificateless CPPA scheme for VANETs without using bilinear pairing and map-to-point hash operations.

### 1.1. Our Contributions

This paper proposes a practical certificateless conditional privacy preserving authentication (PCPA) scheme for VANETs. To summarize, the major contributions of this paper are as follows:

- A certificateless signature with message recovery (CLS-MR), which is proved to be secure under the assumption of elliptic curve discrete logarithm (ECDL) in the random oracle, is proposed based on certificateless cryptography [16] and elliptic curve cryptography (ECC) [17,18]. This is of independent interest.
- A practical certificateless conditional privacy preserving authentication (PCPA) scheme for VANETs is proposed based on CLS-MR. The security analysis and comparison indicate that PCPA satisfies all security and privacy requirements.
- The performance in computation and communication cost is evaluated through quantitative calculations. Experimental results depict that PCPA is more efficient than other schemes in [12,13,14,15].
- An extensive simulation is performed and the results display that PCPA is more feasible and achieves the low average message delay and message loss ratio.

### 1.2. Organization

Organization of this paper is demonstrated as follows: in Section 2, we survey the related work about CPPA in VANETs. In Section 3, the preliminaries are introduced. We present the concrete PCPA scheme for V2I communication in Section 4. Section 5 analyzes the security of the proposed scheme. Section 6 conducts the performance evaluations and experimental simulation results. Finally, Section 7 concludes the paper.

## 2. Related Works

A lot of researchers have put great efforts on authentication schemes aimed to achieve security, privacy and efficiency. These schemes are roughly classified into three categories: PKI-based authentication schemes, ID-based authentication schemes, and certificateless authentication schemes.

In the first category, the anonymous certificates are used to hidden the vehicle’s real identities. In 2004, Hubaux et al. [4] claimed that the PKI technology could be used to address the security and privacy preserving problems in VANETs. In 2007, Raya and Hubaux [1], based on PKI and anonymous certificates, put forward an anonymous authentication scheme for VANETs. In this scheme, each vehicle needs to preload lots of anonymous public/private key pairs and the corresponding public key certificates. In this case, the vehicles need a large storage spaces and a huge verification overhead. Furthermore, a trusted authority (TA) will generate a large certificate revocation list (CRL), making the revocation mechanism very inefficient. In 2008, Lu et al. [10] constructed an efficient conditional privacy preserving (ECPP) mechanism for VANETs, to solve the storage space problem and the CRL growth problem in [11]. Zhang et al. [19] proposed a message authentication scheme based k-anonymity approach and hash message authentication code to achieve the privacy preserving of the vehicles and low communication cost. However, all the PKI-based authentication schemes for VANETs have a bottleneck problem on the management and storage of certificates.

ID-based authentication schemes for VANETs have been proposed so as to solve the problems mentioned above. Incorporating the ID-based cryptography [20], Zhang et al. [11,21] proposed ID-based CPPA schemes supporting batch verification based on bilinear pairing for VANETs. In these schemes, the RSU and the vehicle utilize the pseudo-identity information as the public keys, while the private keys are generated by a trusted third party, namely, the private key generator (PKG). Thus, these schemes avoid the requirements of certificate storage in the entities, and alleviate the certificate management of PKI. Furthermore, the schemes achieve low verification cost because of batch message verification, which allows a large number of messages to be verified simultaneously. In 2009, based on binary authentication tree, an ID-based authentication scheme for V2I communication is proposed by Jiang et al. [22]. This scheme meets the security and privacy requirements, and achieves high efficiency in VANETs. In 2011, Chim et al. [23] pointed out that the schemes proposed in [11,21] were insecure against impersonation and anti-traceability attacks, then constructed a secure communication scheme for VANETs. Based on bilinear pairing, Huang et al. [24] presented a new authentication scheme for VANETs that not only is efficient in performances, but also provides conditional privacy to the vehicles. Based on the pseudo-identity-based signature, Shim [25] proposed an ID-based CPPA scheme for VANETs. In 2013, Shim [26] and Li et al. [27] pointed out that the schemes in [11,22] were insecure against the security attacks, and then established the improved ID-based authentication schemes. Horng et al. [28] showed that scheme in [23] is not secure against impersonation attack and proposed a secure scheme to make up for the security flaw in [23]. In 2014, Zhang et al. [29], aiming at the weakness mentioned in [27], constructed an improved ID-based CPPA scheme for VANETs. Liu et al. [30] indicated that the underlying ID-based signature scheme in [25] was unable to reach an acceptable security level, and thus the corresponding Coron’s technique authentication scheme suffers from a modification attack. In 2015, Bayat et al. [31] further pointed out the security flaws in [27] and designed a new scheme. Based on bilinear pairing, ID-based authentication schemes [32,33,34,35,36] were proposed, which are capable of guaranteeing the security and privacy requirements in VANETs. However, the performance of such schemes is not satisfactory because bilinear pairing operations should be used to implement authentication in VANETs. Based on the ECC, efficient ID-based authentication schemes for VANETs were proposed in [37,38,39,40,41,42,43], where bilinear pairing operations and map-to-hash operations are not applied. They achieve high efficiency in terms of computation and communication cost. Although ID-based authentication schemes eliminate the certificates, simplify the key management and reduce the storage overhead, they are confronted with the inherent key escrow challenge. That is to say, PKG has the knowledge on the private keys of all vehicles and RSUs. It appears that this condition may be excessively strong and not appropriate for VANETs.

To solve the key escrow problem in ID-based authentication schemes, certificateless authentication schemes have been proposed for VANETs. Horng et al. [12], based on certificateless cryptography [16], put forward a secure certificateless CPPA scheme. In this scheme, only the partial private key of the users (RSU and Vehicle) is generated by a trusted party, namely, the Key Generator Center (KGC). A secret value is picked by the user itself, and combines the partial private key to form the private key. Therefore, the KGC has no the private key s of all users. Moreover, in the certificateless CPPA scheme, public key certificates are not needed to guarantee the authenticity of public keys. In 2016, Li et al. [13] found that the scheme in [12] was not secure against a malicious-but-passive KGC under the existing security model. In other words, KGC may maliciously implant a trapdoor in the public system parameters and attempts to forge a signature without the vehicle’s private key. Based on bilinear pairing, an efficient certificateless aggregate signature scheme for VANETs was put forward by Malhi et al. [14], which achieves low computation cost s in verification phase. In 2018, Kumar et al. [15] demonstrated that the scheme in [14] was vulnerable to malicious KGC attack and proposed an improved scheme for VANETs, which was able to eliminate the security flaws of scheme in [14] and achieved the same performances.

Upon reviewing the literature, the aforementioned schemes have different problems. The PKI-based schemes suffer from the high cost of certificate management on CA, in which the vehicles could easily disrupt the service of VANETs. As for ID-based schemes, a key escrow problem is inevitable and incurs the security of VANETs. Until now, the existing certificateless schemes solve the above problems in PKI-based and ID-based schemes but are still not efficient and suitable to VANETs because of the huge computation overhead and communication cost.

The proposed scheme had addressed the aforementioned issues simultaneously based on the ECC. It neither requires the certificate management, nor the involves key escrow problem. Moreover, the proposed scheme does not use bilinear pairing and map-to-point hash operations, which achieves outstanding performances and is more suitable for VANETs than other schemes.

## 3. Preliminaries

The elliptic curves and related problem, system model, security requirement and cryptographic primitive used as building blocks are introduced in this section. For readability, the notations adopted in the present paper are listed in Table 1.

### 3.1. Elliptic Curves

Miller [17] and Koblitz [18] first proposed the concept of elliptic curve cryptography (ECC).

Let $F_{p}$ be a finite field with a large prime *p*. The elliptic curve *E* over $F_{p}$ is defined as the set of an infinity point *O* and all points P=(x,y) that meet the equation $y^{2}=x^{3}+ax+b\;\left(\mathrm{mod}p\right)$, where the discriminant $\Delta =4a^{3}+27b^{2}\neq 0$ and $a,b\in F_{p}$. The elliptic curve *E* forms an additive cyclic group G under the operation of point addition P+Q=R. Scalar multiplication operation over $F_{p}$ is expressed as kP=P+P+···+P(ktimes). The hard problems based on ECC are shown as follows:

- Elliptic curve discrete logarithm (ECDL) problem: Given two random points P,Z=yP∈G, find an integer *x*, such that Z=xP.
- Elliptic curve discrete logarithm (ECDL) assumption problem: There are no polynomial-time algorithms to solve the ECDL problem with non-negligible probability.
- Elliptic curve computational Differ-Hellman (ECCDH) problem: For unknown x,y integers and the given two random points R=xP,Z=yP∈G, calculate the point xyP .
- Elliptic curve computational Differ-Hellman (ECCDH) assumption: There are no polynomial-time algorithms to solve the ECCDH problem with non-negligible probability.

### 3.2. System Model

**Figure 1:**
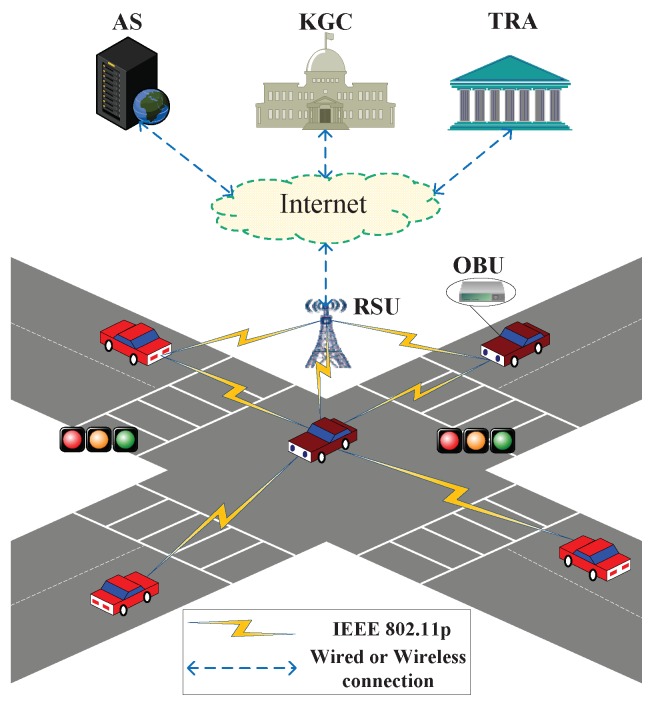
System model.

The system model of the proposed scheme is shown in Figure 1. As is shown in Figure 1, the system is composed of five entities: the Key Generator Center (KGC), the Trace Authority (TRA), the Application Servers (AS), the RSU, and the OBU.

KGC: It is in charge of calculating system parameters and preloading them on RSUs and OBUs in offline mode. In addition, it also produces and distributes the partial private keys for RSUs and OBUs. The KGC is assumed to be a trusted third party with sufficient storage space and computing power.

TRA: It is used for the registration of RSUs and OBUs. It can trace messages to their source and disclose the vehicles’ real identity. Similarly, the TRA is assumed to be a trusted third party with sufficient storage space and computing power.

AS: It is a safety-related application server, like a traffic-data analysis center or traffic manage center. It first gathers the traffic-related messages including current location, time, traffic accidents from RSUs, and then conducts further analysis and/or provides feedback to them. The AS communicates with KGC, TRA and RSUs via the wired channel.

RSU: It is located along the roadside and is used for verifying the authenticity and integrity of messages and processing them locally or forwarding them to TAs or AS when received the messages from OBUs. The RSU communicates with the vehicle in a certain coverage region by a wireless channel and communicates with KGC, TRA and AS via a secure wired channel.

OBU: It is installed on the vehicle to communicate with other vehicles and RSUs for sharing traffic-related status information like speed, direction, and position through the Dedicated Short Range Communication (DSRC) [2,3]. Generally, the OBU is assumed to have less computation power than RSU.

### 3.3. Security Requirements

In V2I communication, the following security requirements need to be satisfied in the proposed scheme.

**Authentication and message integrity**: The message receiver (RSU) should be able to verify the legality of the vehicle efficiently in the system and detect any modification of the received message.

**Identity privacy preserving**: Any entity should not identify or trace the vehicle’s real identity by analyzing the received messages.

**Traceability**: The generator of any mistake message should be traceable. TRA should be able to disclose the real identity of any malicious vehicle, which has broadcasted forged messages to other vehicles in order to disrupt the traffic.

**Unlinkability**: Apart from TRA, neither should the RSU nor the malicious vehicle be able to determine whether two messages are from the same vehicle.

**Key escrow resilience**: KGC, a semi-trusted party, should not impersonate legitimate vehicle to generate a valid signature using the vehicle’s private key.

**Role separation**: Two trusted authorities exist in the proposed scheme, i.e., KGC and TRA. KGC is working for creating the vehicle’s partial private key on the pseudo identity. TRA is responsible for producing the pseudo identities and tracing the vehicle’s real identity.

**Resistance to attack**: The proposed scheme should resist various of popular attacks such as the replay attack, the modification attack, the impersonation attack, and the man-in-the-middle attack in VANETs.

### 3.4. CLS-MR

The CLS-MR includes the following algorithms: setup, partial-private-key-extract, set-secret-value, set-private-key, set-public-key, sign, and verify.

- **Setup**: Given a security parameter *k*, the KGC generates a group G of the prime order *q* based on an elliptic curve *E* defined over a finite field $F_{p}$, where P∈G is a generator. The KGC randomly chooses $s\in Z_{q}^{*}$ and computes $P_{pub}=sP$. The KGC also chooses hash functions $H_{1},H_{2},H_{3}:{\left\{0,1\right\}}^{*}\to Z_{q}^{*}$, $F_{1}:{\left\{0,1\right\}}^{l_{2}}\to {\left\{0,1\right\}}^{l_{1}}$ and $F_{2}:{\left\{0,1\right\}}^{l_{1}}\to {\left\{0,1\right\}}^{l_{2}}$, where $l_{1}$ and $l_{1}$ are positive integers such that $l_{1}+l_{2}=\left|q\right|$. The system parameter is $params=\{F_{p},G,q,P,P_{pub},H_{1},H_{2},H_{3},F_{1},F_{2},l_{1},l_{2}\}$ and the master key is *s* .
- **Partial-Private-Key-Extract**: Given params and an identity $ID_{i}$, the KGC chooses at random $r_{i}\in Z_{q}^{*}$ and computes
  - $R_{i}=r_{i}P$,
  - $h_{1i}=H_{1}\left(ID_{i},R_{i}\right)$,
  - $d_{i}=r_{i}+h_{1i}s$.
  The partial private key for $ID_{i}$ is $PPK_{i}=\left\{R_{i},d_{i}\right\}$. The KGC securely returns $PPK_{i}$ to the user.
- **Set-Secret-Value**: The user $ID_{i}$ picks a random number $x_{i}\in Z_{q}^{*}$ as its secret value.
- **Set-Private-Key**: The private key of user $ID_{i}$ is $SK_{i}=\left\{d_{i},x_{i}\right\}$.
- **Set-Public-Key**: Given params and the user’s secret value $x_{i}$, the user $ID_{i}$ computes $P_{i}=x_{i}P$ and sets $PK_{i}=\left\{R_{i},P_{i}\right\}$ as its public key.
- **Sign**: Given params, private key $\left\{d_{i},x_{i}\right\}$ for the user $ID_{i}$ under $\left\{R_{i},P_{i}\right\}$ and a message $m\in {\left\{0,1\right\}}^{l_{2}}$, the user $ID_{i}$ picks a random number $t_{i}\in Z_{q}^{*}$ and computes
  - $f=F_{1}\left(m\right)\left|\right|F_{2}\left(F_{1}\left(m\right)\right)⊕m$,
  - $u_{i}=f⊕\left(t_{i}P\right)$,
  - $h_{2i}=H_{2}\left(ID_{i},P_{pub},P_{i}\right)$,
  - $h_{3i}=H_{3}\left(ID_{i},P_{pub},R_{i},u_{i}\right)$,
  - $v_{i}=t_{i}+h_{2i}x_{i}+h_{3i}d_{i}$.
  Finally, the signature on *m* for $ID_{i}$ is $\sigma_{i}=\left\{u_{i},v_{i}\right\}$.
- **Verify**: Given params , the public key $\left\{R_{i},P_{i}\right\}$, the user’s identity $ID_{i}$ and the signature $\sigma_{i}$, any verifier recovers the message and checks the validity of signature. To recover message *m*, the verifier computes
  - $h_{1i}=H_{1}\left(R_{i},ID_{i}\right)$,
  - $h_{2i}=H_{2}\left(ID_{i},P_{pub},P_{i}\right)$,
  - $h_{3i}=H_{3}\left(ID_{i},P_{pub},R_{i},u_{i}\right)$,
  - $f=u_{i}⊕\left(v_{i}P-h_{2i}P_{i}-h_{3i}R_{i}-h_{3i}h_{1i}P_{pub}\right)$,
  - $m={\left[f\right]}_{l_{2}}⊕F_{2}{(}_{l_{1}}\left[f\right])$ where ⊕ is exclusive or operation, ${}_{l_{1}}\left[f\right]$ and ${\left[f\right]}_{l_{2}}$ are the most significant $l_{1}$-bit of *f* and the least significant $l_{2}$-bit of *f*, respectively.
  **Correctness**:
  Given a signature $\sigma_{i}=\left\{u_{i},v_{i}\right\}$ for $ID_{i}$ under $\left\{R_{i},P_{i}\right\}$, compute $h_{1i}=H_{1}\left(ID_{i},R_{i}\right)$, $h_{2i}=H_{2}\left(ID_{i},P_{pub},P_{i}\right)$, $h_{3i}=H_{3}\left(ID_{i},P_{pub},R_{i},u_{i}\right)$, and
  $$\begin{array}{c} \;u_{i}⊕\left(v_{i}P-h_{2i}P_{i}-h_{3i}R_{i}-h_{3i}h_{1i}P_{pub}\right)\\ =\left[f⊕\left(t_{i}P\right)\right]⊕\left[\left(t_{i}+h_{2i}x_{i}+h_{3i}d_{i}\right)P-h_{2i}P_{i}-h_{3i}R_{i}-h_{3i}h_{1i}P_{pub}\right]\\ =\left[f⊕\left(t_{i}P\right)\right]⊕\left[t_{i}P+h_{2i}\left(x_{i}P\right)+h_{3i}\left(r_{i}+h_{1i}s\right)P-h_{2i}P_{i}-h_{3i}R_{i}-h_{3i}h_{1i}P_{pub}\right]\\ =f. \end{array}$$
  Then, one can recover
  $$\begin{array}{c} m={\left[f\right]}_{l_{2}}⊕F_{2}{(}_{l_{1}}\left[f\right])\\ \;\;\;\;\;=[F_{1}\left(m\right)||F_{2}\left(F_{1}\left(m\right)\right){⊕m]}_{l_{2}}⊕F_{2}{(}_{l_{1}}[F_{1}\left(m\right)||F_{2}\left(F_{1}\left(m\right)\right)⊕m])\\ \;\;\;\;\;=F_{2}\left(F_{1}\left(m\right)\right)⊕m⊕F_{2}\left(F_{1}\left(m\right)\right)\\ \;\;\;\;\;=m. \end{array}$$

### 3.5. Security Proof

According to certificateless cryptography [16], two types of adversaries, i.e., Type I adversary $A_{1}$ and Type II adversary $A_{2}$, are considered in CLS-MR. The adversary $A_{1}$ models an outside adversary and acts as a malicious third party while the adversary $A_{2}$ models an inside adversary and serves as a malicious-but-passive KGC.

- **Type I adversary $A_{1}$**: The adversary $A_{1}$ is not in possession of the master key, but is capable of replacing the public key of the user with a value chosen by itself.
- **Type II adversary $A_{2}$**: The adversary $A_{2}$ is in possession of the master key, but cannot replace the public key of the user.

The formal security model of CLS-RM is depicted in detail in [16].

**Theorem** **1.** *The proposed CLS-MR is existentially unforgeable under the ECDL assumption in the random oracle model.*

**Proof.**  Theorem 1 is proved according to Lemma 1 and Lemma 2 listed below. ☐

**Lemma** **1.** *In the random oracle model, CLS-MR is existential unforgeable against Type I adversary $A_{1}$ under the ECDL assumption.*

**Lemma** **2.** *In the random oracle model, CLS-MR is existential unforgeable against Type II adversary $A_{2}$ under the ECDL assumption.*

The security proof of Lemma 1 and Lemma 2 can be found in the appendix.

## 4. The Proposed Scheme

This section proposes a practical certificateless conditional privacy-preserving authentication (PCPA) scheme for VANETs based on CLS-MR. Specifically, the proposed scheme includes system initialization, pseudo identity generation and partial private key extraction, public/private key generation and message signing, and message verification phases.

### 4.1. System Initialization

The system initialization, which is carried out by TAs (KGC and TRA), is to produce system parameters for all RSUs and OBUs. The following steps are performed in this phase:

(1) The TAs randomly choose a prime p, an elliptic curve *E* over the finite field $F_{p}$, which is defined by the equation $y^{2}=x^{3}+ax+b\;\mathrm{mod}\;p$, where $4a^{3}+27b^{2}\neq 0$ and $a,b\in F_{p}$ .
(2) The TAs pick a group G of prime order *q* based on *E* and denote P∈G a generator.
(3) The KGC calculates its public key $P_{pub}\;=\;sP$, where $s\in Z_{q}^{*}$ is the master key for partial private key generation.
(4) The TRA chooses a random number $t\in Z_{q}^{*}$ as the master key for identity traceability and computes $T_{pub}=tP$ .
(5) The TAs choose hash functions: $H:{\left\{0,1\right\}}^{*}\to Z_{q}^{*}$, $H_{1}:{\left\{0,1\right\}}^{*}\to Z_{q}^{*}$, $H_{2}:{\left\{0,1\right\}}^{*}\to Z_{q}^{*}$, $H_{3}:{\left\{0,1\right\}}^{*}\to Z_{q}^{*}$, $F_{1}:{\left\{0,1\right\}}^{l_{2}}\to {\left\{0,1\right\}}^{l_{1}}$ and $F_{2}:{\left\{0,1\right\}}^{l_{1}}\to {\left\{0,1\right\}}^{l_{2}}$, where $l_{1}$ and $l_{1}$ are positive integers such that $l_{1}+l_{2}=\left|q\right|$.

The TAs publish the system parameters $\left\{p,q,G,P,P_{pub},T_{pub},H,H_{1},H_{2},H_{3},F_{1},F_{2}\right\}$ and send them to all RSUs and vehicles (OBUs). Here, the system parameters are preloaded into the all vehicles’ tamper-proof devices (TPD) for VANETs. The master keys *s* and *t* are kept secretly by KGC and TRA, respectively.

### 4.2. Pseudo Identity Generation and Partial Private Key Extraction

This phase is performed between the TAs (TRA and KGC) and the vehicles. Receiving the real identity $RID_{i}$ from $V_{i}$, where $RID_{i}$ uniquely identifies the vehicle $V_{i}$, the KGC calculates partial private keys on them after the TRA generates pseudo identities for the vehicle $V_{i}$. Then, the partial private keys and pseudo identities are preloaded in TPD of vehicle $V_{i}$. The details of this phase are as follows:

(1) The vehicle $V_{i}$ sends the real identity $RID_{i}$ to the TRA in secure mode.
(2) Upon receiving the real identity $RID_{i}$, the TRA randomly chooses $w_{i}\in Z_{q}^{*}$ and computes
  - $PID_{i,1}=w_{i}P$,
  - $PID_{i,2}=RID_{i}⊕H\left(w_{i}T_{pub},T_{i}\right)$, where $T_{i}$ defines the valid period of the pseudo identity $PID_{i}$.
  Then, a pseudo identity $PID_{i}=\left\{PID_{i,1},PID_{i,2},T_{i}\right\}$ is transmitted to the KGC via a secure way.
(3) When receiving the pseudo identity $PID_{i}=\left\{PID_{i,1},PID_{i,2},T_{i}\right\}$, the KGC randomly chooses $r_{i}\in Z_{q}^{*}$ and calculates the partial private key $PPK_{i}=\left\{R_{i},d_{i}\right\}$ using the master key *s* where
  - $R_{i}=r_{i}P$,
  - $d_{i}=r_{i}+sH_{1}\left(PID_{i},R_{i},P_{pub},T_{pub}\right)$.
(4) After that, the KGC sends the partial private key and pseudo identity $\left\{PPK_{i},PID_{i}\right\}$ to the vehicle $V_{i}$.

### 4.3. Public/Private Key Generation and Message Signing

During this phase, the vehicle $V_{i}$ generates public/private key and signs messages. Then, the vehicle $V_{i}$ broadcasts a final message, including the pseudo identity, public key, timestamp, and signature, to nearby RSUs. The details of this phase are as follows:

(1) The vehicle $V_{i}$ randomly picks $x_{i}\in Z_{q}^{*}$ as the secret value and computes $P_{i}\;=\;x_{i}P$. Then, the vehicle $V_{i}$’s private key is $SK_{i}=\left\{d_{i},x_{i}\right\}$ and the public key is $PK_{i}=\left\{R_{i},P_{i}\right\}$.
(2) The vehicle $V_{i}$ randomly chooses a pseudo identity $PID_{i}$ from its storage and a current timestamp $ct_{i}$, which is used to ensure the freshness of message so as to resist the replay attack. Given a traffic-related message $m_{i}\in {\left\{0,1\right\}}^{l_{2}}$, the vehicle $V_{i}$ randomly picks $t_{i}\in Z_{q}^{*}$, and calculates
  - $f=F_{1}\left(m_{i}\right)\left|\right|F_{2}\left(F_{1}\left(m_{i}\right)\right)⊕m_{i}$,
  - $u_{i}=f⊕\left(t_{i}P\right)$,
  - $h_{2i}=H_{2}\left(PID_{i},P_{pub},T_{pub},P_{i},ct_{i}\right)$,
  - $h_{3i}=H_{3}\left(PID_{i},P_{pub},T_{pub},R_{i},u_{i},ct_{i}\right)$,
  - $v_{i}=t_{i}+h_{2i}x_{i}+h_{3i}d_{i}$.

The signature of a traffic-related message $m_{i}$ is $\left\{u_{i},v_{i}\right\}$. Then, the vehicle $V_{i}$ broadcasts the final message $M_{i}=\left\{PID_{i},PK_{i},ct_{i},u_{i},v_{i}\right\}$ to nearby RSUs.

### 4.4. Message Verification

In this phase, after receiving the final message $\left\{PID_{i},PK_{i},ct_{i},u_{i},v_{i}\right\}$, the verifier (RSU) recovers the messages and checks the validity of the signature. Based on this, it is a guarantee that the corresponding vehicle cannot broadcast false messages or masquerading as other legal vehicles. This phase is described as follows:

(1) The verifier checks whether $T_{i}$ is valid and $ct_{i}$ is fresh. If $T_{i}$ is not valid or $ct_{i}$ is not fresh, the message will be rejected.
(2) The verifier computes
  - $h_{1i}=H_{1}\left(PID_{i},R_{i},T_{pub},P_{pub}\right)$,
  - $h_{2i}=H_{2}\left(PID_{i},P_{pub},T_{pub},P_{i},ct_{i}\right)$,
  - $h_{3i}=H_{3}\left(PID_{i},P_{pub},T_{pub},R_{i},u_{i},ct_{i}\right)$,
  - $f_{i}=u_{i}⊕\left(v_{i}P-h_{2i}P_{i}-h_{3i}R_{i}-h_{3i}h_{1i}P_{pub}\right)$,
  - $m_{i}={\left[f_{i}\right]}_{l_{2}}⊕F_{2}{(}_{l_{1}}\left[f_{i}\right])$.
(3) Checks whether ${}_{l_{1}}\left[f_{i}\right]=F_{1}\left(m_{i}\right)$.

## 5. Security Analysis

In this section, an analysis on the security of the proposed scheme as well as its comparison with the latest schemes is conducted.

**Authentication and message integrity**: To ensure the authentication and message integrity, a new CLS-MR scheme is employed in the proposed PCPA. According to Theorem 1, the underlying CLS-MR is secure against adaptive chosen message and identity attacks under the ECDL assumption in the random oracle model. Through a Message Verification algorithm, a verifier (RSU) can confirm the validity and integrity of $\left\{PID_{i},PK_{i},ct_{i},u_{i},v_{i}\right\}$. That is to say, any polynomial-time adversary is unable to forge or modify a valid signature. Therefore, the message integrity and authentication can be ensured in the proposed scheme.

**Identity privacy preserving**: According to the description of the proposed scheme, the real identity $RID_{i}$ of the vehicle $V_{i}$ is only included in random pseudo identity $PID_{i}=\left\{PID_{i1},PID_{i2},T_{i}\right\}$, where $PID_{i1}=w_{i}P$, $PID_{i,2}=RID_{i}⊕H\left(w_{i}T_{pub},T_{i}\right)$ and $T_{pub}=tP$. To extract the vehicle ${V_{i}}^{\prime }s$ real identity $RID_{i}$, the adversary has to compute $RID_{i}=PID_{i,2}⊕H_{1}\left(w_{i}T_{pub},T_{i}\right)=PID_{i,2}⊕H_{1}\left(w_{i}\cdot t\cdot P,T_{i}\right)$. However, without knowing $w_{i}$ and *t*, it is impossible for any adversary to obtain $RID_{i}$ as it is an instance of a ECCDH problem to solve $w_{i}\cdot t\cdot P$. Therefore, the identity privacy preserving can be ensured in the proposed scheme.

**Traceability**: According to the description of the proposed scheme, the TRA can use its own master key *t* to compute $t\cdot PID_{i1}=t\cdot w_{i}\cdot P=w_{i}\cdot t\cdot P=w_{i}\cdot T_{pub}$ and $RID_{i}=PID_{i,2}⊕H_{1}\left(w_{i}T_{pub},T_{i}\right)$. TRA can extract the real identity $RID_{i}$ from a pseudo identity $PID_{i}=\left\{PID_{i,1},PID_{i,2},T_{i}\right\}$ involved in the broadcast messages. Therefore, the proposed scheme satisfies the traceability.

**Unlinkability**: According to the description of the proposed scheme, the TRA, KGC, and the vehicle randomly choose $w_{i}\in Z_{q}^{*}$, $r_{i}\in Z_{q}^{*}$ and $t_{i}\in Z_{q}^{*}$ respectively, and generates $\left\{PID_{i},PK_{i},ct_{i},u_{i},v_{i}\right\}$, where $PID_{i1}=w_{i}P$, $PID_{i,2}=RID_{i}⊕H_{1}\left(w_{i}T_{pub},T_{i}\right)$, $PID_{i}=\left\{PID_{i,1},PID_{i,2},T_{i}\right\}$, $R_{i}=r_{i}P$, $d_{i}=r_{i}+sH_{1}\left(PID_{i},R_{i},T_{pub},P_{pub}\right)$, $f=F_{1}\left(m\right)\left|\right|F_{2}\left(F_{1}\left(m\right)\right)⊕m$, $u_{i}=f⊕\left(t_{i}P\right)$ and $v_{i}=t_{i}+x_{i}H_{2}\left(PID_{i},P_{pub},T_{pub},P_{i},ct_{i}\right)+d_{i}H_{3}\left(PID_{i},P_{pub},T_{pub},R_{i},u_{i},ct_{i}\right)$. Due to the randomness of $w_{i}$, $r_{i}$ and $t_{i}$, any adversary is unable to link two messages sent from the same vehicle or two anonymous pseudo identities, through which the unlinkability of the proposed scheme is satisfied.

**Role separation**: According to the description of the proposed scheme, there are two trusted authorities with different functions, i.e., TRA and KGC. The real identity of a vehicle can only be revealed by TRA rather than KGC by using the master key *t*. Here, *t* have to be well safeguarded for the vehicle’s privacy preserving. However, there is no need to give strong protection to the master key *s* of KGC, since no adversaries can generate a valid signature without the vehicle’s secret value. Therefore, the role separation can be provided in the proposed scheme.

**Key escrow resilience**: According to the Lemma 2, the malicious KGC cannot impersonate a vehicle successfully under the ECDLP assumption. The basic reason is that the vehicle $V_{i}$ calculates the secret value $x_{i}$ itself, and it cannot be accessed by the KGC. Therefore, the key escrow resilience is satisfied in the proposed scheme.

**Resistance to attacks**: The proposed scheme is secure against the main attacks of network. The details are as follows:

- **Replay attack**: It can be known from the description of the proposed scheme, the timestamp $ct_{i}$ is included in $\left\{PID_{i},PK_{i},ct_{i},u_{i},v_{i}\right\}$, which ensures the message freshness to guards against the replay attacks. This requires loose synchronization of the clocks, which could be provided by widely used GPS devices.
- **Modification attack**: Following the depiction of the proposed scheme, we realized that $\left\{u_{i},v_{i}\right\}$ is a signature of the traffic-related message $m_{i}$ under $\left\{PID_{i},PK_{i},ct_{i}\right\}$. Based on the CLS-MR and Theorem 1, any polynomial adversary can not forge a valid signature and RSU can find any modification on $\left\{PID_{i},PK_{i},ct_{i},u_{i},v_{i}\right\}$ by the Message Verification algorithm.
- **Impersonation attack**: It can be known from Theorem 1 that no adversary is able to fabricate the legal message $\left\{PID_{i},PK_{i},ct_{i},u_{i},v_{i}\right\}$ without the vehicle’s private key. By means of the validity checking on the received message, RSU can find the impersonation attack.
- **Man-in-the-middle attack**: As is shown in the analysis on the modification attack, any modification about $\left\{PID_{i},PK_{i},ct_{i},u_{i},v_{i}\right\}$ in transmission can be found.

We compare the security of the proposed PCPA scheme for VANETs with that of the schemes put forwarded by Horng et al. [12], Li et al. [13], Malhi et al. [14], and Kumar et al. [15]. Details on the security comparisons between the proposed scheme and the abovementioned schemes are given in Table 2, where ✓ indicates “satisfy” and ✗ refers to “not satisfy”.

## 6. Performance Evaluation and Simulation

Here, we analyze the computation and communication costs of the proposed PCPA and evaluate its performance with the existing schemes in [12,13,14]. It should be pointed out that the analysis and comparison of Kumar et al.’s scheme [15] are omitted, as it has only made a small change in the signing phase to fix the security flaw in [14]. Moreover, a comprehensive simulation is carried out using simulation of urban mobility (SUMO) [44] and ns-3.26 simulator [45]. SUMO is a traffic simulation tool that can provide the realistic traffic mobility model and ns-3.26 is used for wireless network simulation. Based on the simulations, we give concrete evaluation on average message delay and average message loss ratio in real scenarios.

### 6.1. Computation Cost

The computation cost for the message signing and verification in the proposed scheme is analyzed and the results are compared with those obtained from the schemes put forward by Horng et al. [12], Li et al. [13], and Malhi et al. [14].

For the pairing-based schemes [12,13,14], the symmetric bilinear pairing for the 80-bit security can be defined as follows: $e:G_{1}\times G_{1}\to G_{T}$, where $G_{1}$ is an additive group formed by a generator *P* with the order *q* on a super singular elliptic curve $E:y^{2}=x^{3}+x\;\mathrm{mod}\;p$ with embedding degree 2. *q* is 160-bit Solinas prime number and *p* is 512-bit prime number, which satisfy q·12·r=p+1. For the proposed scheme, the ECC for the same security level can be constructed as follows: G with order *q* is an additive group generated by a point *P* on a non-singular elliptic curve $E:y^{2}=x^{3}+ax+b\;\mathrm{mod}\;p$, where *p*, *q* are two 160-bit prime numbers, a=-3, and *b* is a random 160-bit prime number.

The time cost for performing the cryptographic operations is defined below. Let $T_{p}$ be the time to perform a bilinear pairing operation, $T_{m-bp}$ and $T_{m-ecc}$ be the time to perform a scale multiplication operation in bilinear pairing and ECC, respectively. The time to perform a map-to-point hash function operation is denoted as $T_{mtp}$. Other lightweight operations (point addition, and one-way hash function operation) are not taken into account.

Using the MIRACL Crypto SDK [46], the running time of the above cryptographic operations can be quantified. The experiment is run on Intel Corei5-4590 (Intel Corporation, Santa Clara, CA, USA), 3.3 GHz CPU, 8 gigabytes memory with Windows 7 (Microsoft Corporation, Redmond, WA, USA). The average execution times of those operations are listed in Table 3.

Based on the experiment results, the computation costs of Horng et al.’s scheme [12], Li et al.’s scheme [13], Mahli et al.’s scheme [14] and the proposed PCPA are compared and shown in Table 4.

For the computation cost of one message signing, Horng et al.’s scheme [12] requires two scalar multiplication operations in bilinear pairing. Therefore, the total signing time is 2$T_{m-bp}=7.5540$ ms. Li et al.’s scheme [13] requires one map-to-point hash operation and two scalar multiplication operations in bilinear pairing. Thus, the total signing time is $T_{mtp}+2T_{m-bp}=17.2592$ ms. Malhi et al.’s scheme [14] requires four scalar multiplication operations in bilinear pairing. Thus, the total signing time is 4$T_{m-bp}=15.1080$ ms. The proposed scheme requires one scalar multiplication operation in ECC. Thus, the total signing time is 1$T_{m-ecc}$ = 0.8310 ms.

For the computation cost of one message verification, Horng et al.’s scheme [12] requires one map-to-point hash operation, one scalar multiplication operation in bilinear pairing and three bilinear pairing operations. Thus, the total verification time is $T_{mtp}$ + $T_{m-bp}$ + 3$T_{p}$ = 40.7195 ms. Li et al.’s scheme [13] requires two map-to-point hash operations, one scalar multiplication operation in bilinear pairing and three bilinear pairing operations. Thus, the total verification time is 2$T_{mtp}$ + $T_{m-bp}$ + 3$T_{p}$ = 50.4247 ms. Mahli et al.’s scheme [14] requires three scalar multiplication operations in bilinear pairing and three bilinear pairing operations. Thus, the total verification time is 3$T_{m-bp}$ + 3$T_{p}$ = 38.5683 ms. The proposed scheme requires four scalar multiplication operations in ECC. Therefore, the total verification time is 4$T_{m-ecc}$ = 3.3240 ms.

**Figure 2:**
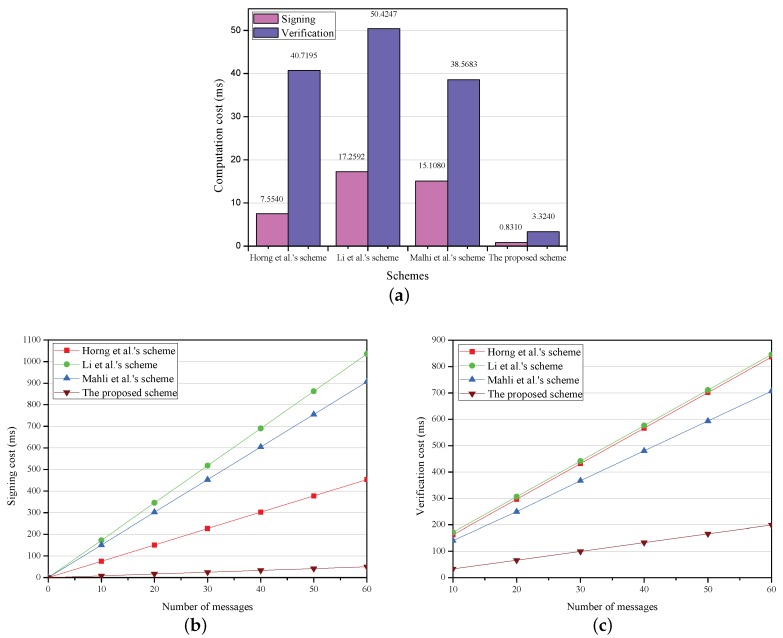
Computation cost. (**a**) computation cost in one message signing and verification; (**b**) signing cost versus number of messages; (**c**) verification cost versus number of messages.

Figure 2 clearly indicates the computation cost for one message and that with an increasing number of messages, respectively. As is shown in Table 4 and Figure 2a, the computation cost of a message signing is 0.8310 ms in the proposed scheme, which decreases by 88.9%, 95.2% and 94.5% compared with those in [12,13,14], respectively. In terms of the computation overhead of one message verification, the proposed scheme needs 3.3240 ms, which decreases by 91.8%, 93.4% and 91.4% compared with those in [12,13,14], respectively.

To obtain the computation cost of multiple (*n*) messages signing, the computation delay of one message signing should be repeated *n* times. Therefore, the computation costs of *n* messages signing in [12,13,14] and the proposed scheme are 7.5540n ms, 17.2592n ms, 15.1080n ms, and 0.8310n ms, respectively.

For computation cost of multiply (*n*) messages verification, Horng et al.’s scheme [12] requires *n* map-to-point hash operations, *n* scalar multiplication operations in bilinear pairing and three bilinear pairing operations. Thus, the total verification time is $nT_{mtp}$ + $nT_{m-bp}$ + 3$T_{p}$ = 13.4822*n* + 27.2373 ms. Li et al.’s scheme [13] requires (n+1) map-to-point hash operations, *n* scalar multiplication operations in bilinear pairing and three bilinear pairing operations. Thus, the total verification time is $\left(n+1\right)T_{mtp}$ + $nT_{m-bp}$ + 3$T_{p}$ = 13.4822n+36.9425 ms. Mahli et al.’s scheme [14] requires 3n scalar multiplication operations in bilinear pairing and three bilinear pairing operations. Thus, the total verification time is 3$nT_{m-bp}$ + 3$T_{p}$ = 11.3310n+27.2373 ms. The proposed scheme requires 4n scalar multiplication operations in ECC. Therefore, the total verification time is 4$nT_{m-ecc}$ = 3.3240n ms.

It is known from Figure 2b,c that the signing cost together with verification cost grows linearly with the increase of the number of messages. In addition, the proposed scheme has the lowest slope. As is shown in Figure 2b, when n=60, the signing costs of the schemes in [12,13,14] and the proposed scheme respectively are 453.2400 ms, 1035.5520 ms, 906.4800 ms, 49.8600 ms. As is shown in Figure 2c, the verification costs of the schemes in [12,13,14] and the proposed scheme respectively are 162.0593 ms, 171.7645 ms, 140.5473 ms, and 33.2400 ms when n=10, and 836.1693 ms, 845.8745 ms, 707.0973 ms, and 199.4400 ms when n=60.

Therefore, the proposed PCPA achieves lower computation cost than the schemes in [12,13,14] in the signing and verification phases, regardless of the number of messages.

### 6.2. Communication Cost

In this subsection, the communication costs of Horng et al.’s scheme [12], Li et al.’s scheme [13], Malhi et al.’s scheme [14] and the proposed scheme are evaluated. In V2I communication, the communication cost refers to the size of message transmitted from a vehicle (OBU) to an RSU.

As is mentioned above, the length of *q* is 160 bits and that of *p* is 512 bits, so the length of elements in G and $G_{1}$, respectively, are 20 bytes and 64 bytes. Assuming that the output length of general one-way hash function is 160 bits (20 bytes), and the length of the timestamp is 32 bits (4 bytes). According to IEEE Trial-Use standard [47] for VANETs security, the length of the traffic-related message is 67 bytes. The comparison of communication cost is shown in Table 5 and analyzed as follows.

In [12,13], $\left\{M_{i},PID_{i},P_{i},ct_{i},R_{i},S_{i}\right\}$ is sent from the vehicle (OBU) to a RSU, where $PID_{i}=\left\{PID_{i,1},PID_{i,2},T_{i}\right\}$, $PID_{i,1}\in G_{1}$, $PID_{i,2}\in Z_{q}$ and $T_{i}$ denotes a timestamp. Thus, the communication cost of these two schemes is 351 bytes as

$$|M_{i}|+|PID_{i}\left|+\right|P_{i}\left|+\right|ct_{i}\left|+\right|R_{i}\left|+\right|S_{i}|=67+88+64+4+64+64=351\;bytes.$$

In [14], $\left\{M_{i},PID_{i},P_{i},U_{i},V_{ijk}\right\}$ is sent from the vehicle (OBU) to a RSU, where $PID_{i}=PS1_{i}\in G_{1}$. Thus, the communication cost of this scheme is 323 bytes as

$$|M_{i}|+|PID_{i}\left|+\right|P_{i}\left|+\right|U_{i}\left|+\right|V_{ijk}|=67+64+64+64+64=323\;bytes.$$

In the proposed PCPA, $\left\{PID_{i},PK_{i},ct_{i},u_{i},v_{i}\right\}$ is sent from the vehicle (OBU) to a RSU, where $PID_{i}=\left\{PID_{i,1},PID_{i,2},T_{i}\right\}$, $PID_{i,1}\in G$, $PID_{i,2}\in Z_{q}$ and $T_{i}$ denotes a timestamp. Thus, the communication cost of the proposed scheme is 195 bytes as

$$|PID_{i}|+|PK_{i}|+|ct_{i}\left|+\right|u_{i}\left|+\right|v_{i}|=44+40+4+20+20=128\;bytes.$$

**Figure 3:**
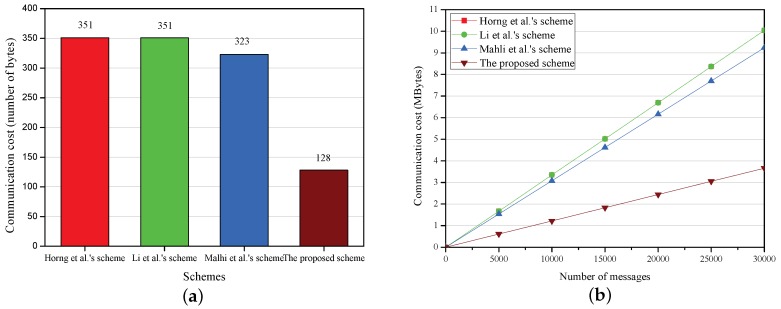
Communication cost. (**a**) communication cost of one message; (**b**) communication cost versus number of messages.

The comparisons on the communication costs of one message and multiply (n) messages is shown in Figure 3. The communication costs increase linearly with the growth of the number of messages in all schemes. The schemes in [12,13] are the same in communication costs. The communication costs of the proposed scheme are the lowest in all schemes, which significantly decreases by 63.5%, 63.5%, and 60.4% compared with those of the schemes in [12,13,14], respectively. When the number of messages is 30,000, the proposed scheme can save 6.38 MB and 5.58 MB bandwidth compared with the schemes [12,13,14], respectively.

### 6.3. Simulation

Exploring SUMO [44] and ns-3.26 [45], we evaluate the performances of the schemes of Horng et al. [12], Li et al. [13], and Malhi et al. [14] as well as the proposed PCPA scheme. The SUMO is used to generate detailed vehicle movement traces by employing models, and then these traces is put into the ns-3.26 simulator to assess the efficiency and applicability of the schemes.

**Figure 4:**
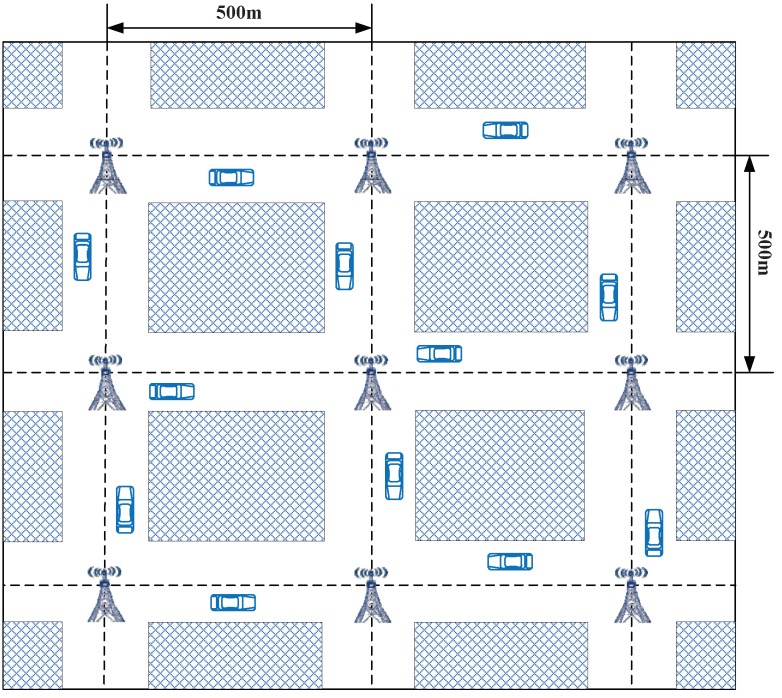
Road scenario for simulation.

The simulation road scenario is shown in Figure 4, in which the RSUs are distributed every 500 m along the road, and each vehicle broadcasts messages every 300 ms. The vehicles are distributed on the road and move to the crossings randomly. The parameters for the simulation are listed in Table 6.

The average message delay (aMD) and average message loss ratio (aMLR) are defined through the notions below:

- $N_{R}$: The number of RSUs within the simulation area.
- $N_{V}$: The number of vehicles within the simulation area.
- $N_{M}^{i}$: The number of messages sent by vehicle $V_{i}$.
- $T_{V_{i}\to RSU_{j},M_{k}}^{S}$: The time for $V_{i}$ sending a message $M_{k}$ to $RSU_{j}$.
- $T_{V_{i}\to RSU_{j},M_{k}}^{R}$: The time for $RSU_{j}$ receiving a message $M_{k}$ from $V_{i}$.
- $T_{avg}^{V}$: The average verification time for each message.
- $N_{A}^{j}$: The number of messages received by $RSU_{j}$ in the media access control (MAC) layer.
- $N_{D}^{j}$: The number of messages dropped by $RSU_{j}$ in the application layer.

Average Message Delay (aMD)

The aMD reflects the average time latency for a message to be received by the RSU after it is generated, which is defined as

$$aMD=\frac{\sum_{i=1}^{N_{V}}\sum_{j=1}^{N_{R}}\sum_{k=1}^{N_{M}^{i}}\left(T_{V_{i}\to RSU_{j},M_{k}}^{R}-T_{V_{i}\to RSU_{j},M_{k}}^{S}\right)}{\sum_{i=1}^{N_{V}}N_{M}^{i}}+T_{avg}^{V}$$.

**Figure 5:**
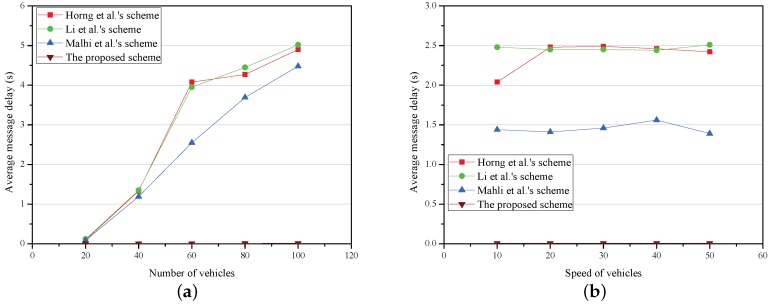
Average message delay. (**a**) average message delay versus number of vehicles; (**b**) average message delay versus speed of vehicles.

Two experiments are conduced to analyze that how aMD with the density and speed of vehicles. The results of simulation are demonstrated in Figure 5.

The relationship between aMD and the number of vehicles is described in Figure 5a, where the number of vehicles varies from 20 to 100, and the average speed of vehicles is approximately 20 m/s (72 km/h). As is shown in Figure 5a, the aMD for RSUs increases with the number of vehicles in all schemes. The aMD is 2.94 s, 2.98 s, 2.40 s and 0.009 s in Horng et al.’s scheme [12], Li et al.’s scheme [13], Mahli et al.’s scheme [14] and the proposed scheme, respectively. In addition, the aMD of the proposed scheme is the lowest, which is slightly influenced by vehicle density.

The relationship between aMD and the speed of vehicles is shown in Figure 5b. The average speed of vehicles varies from 10 to 50 m/s (36 to 180 km/h) and the number of vehicles is 50. Obviously, when the vehicle density is constant, the aMD hardly changes, indicating that it is scarcely affected by the vehicle speed. This is only a theoretical simulation result with no practical implementation.

Average Message Loss Ratio (aMLR) 

The aMLR expresses the ratio of the number of messages dropped to the total number of messages received by the RSUs, which is defined as

$$aMLR=\frac{1}{N_{R}}\sum_{j=1}^{N_{R}}\frac{N_{D}^{j}}{N_{A}^{j}}.$$

**Figure 6:**
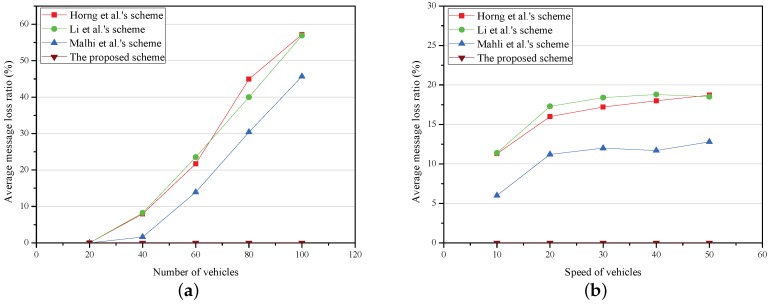
Average message loss ratio. (**a**) average message loss ratio versus number of vehicles; (**b**) average message loss ratio versus speed of vehicles.

Two experiments are conducted to analyze aMLR with the density and speed of vehicles. The results of simulation are shown in Figure 6.

The relationship between aMLR and the number of vehicles is shown in Figure 6a, where the number of vehicles varies from 20 to 100 and the average speed of vehicles is approximately 20 m/s (72 km/h). Under the fixed vehicle speed, when the number of vehicles is larger than 20, the aMLR grows with the number of vehicles in Horng et al.’s scheme [12], Li et al.’s scheme [13] and Malhi et al.’s scheme [14]. Furthermore, the aMLRs respectively hit 57%, 57%, 46% in the schemes of [12,13,14] when the number of vehicles is 100. No matter the density of the vehicles, the aMLR is almost 0.

Figure 6b shows the relationship between aMLR and the speed of vehicles. The speed of vehicles varies from 10 to 50 m/s (36 to 180 km/h) and the number of vehicles is 50. It is easy to see that, when the speed of vehicles is higher than 20 m/s, the aMLRs in the schemes of Horng et al. [12], Li et al. [13], and Malhi et al. [14] are slightly influenced. The aMLR in the proposed scheme is 0% regardless of how the vehicle speed changes.

## 7. Conclusions

In this paper, a new efficient certificateless signature with message recovery (CLS-MR) is first presented. Under the ECDLP assumption, this scheme is secure in the random oracles. Based on the invented CLS-MR, a practical certificateless conditional privacy-preserving authentication (PCPA) scheme for VANETs is put forward. The security analysis indicates that PCPA satisfies the security and privacy-preserving requirements in VANETs. The performance evaluation and comparison show that the PCPA scheme is more efficient in both computation cost and communication cost since it does not employ map-to-point hash function and bilinear pairing operations. Furthermore, the simulation experimental results demonstrate the superiority of PCPA compared to other schemes in average message delay and message loss ratio, and thus PCPA is more suitable for VANETs.

## Figures and Tables

**Table 1:** Notations.

Symbol	Description
p,q	two large prime numbers
$F_{p}$	a finite field over *p*
G	an additive group
*P*	a generator of G
KGC	a key generation center
$\left(P_{pub},s\right)$	KGC’s public key and private key
$H_{1}\left(\cdot \right),H_{2}\left(\cdot \right)$, $H_{3}\left(\cdot \right),H_{4}\left(\cdot \right)$	hash functions: $H_{1},H_{2},H_{3},H_{4}:{\left\{0,1\right\}}^{*}\to Z_{q}^{*}$,
$F_{1}\left(\cdot \right),F_{2}\left(\cdot \right)$	$F_{1}:{\left\{0,1\right\}}^{l_{2}}\to {\left\{0,1\right\}}^{l_{1}}$, $F_{2}:{\left\{0,1\right\}}^{l_{1}}\to {\left\{0,1\right\}}^{l_{2}}$, $l_{1}+l_{2}=\left|q\right|$
$V_{i}$	the *i*-th vehicle
RSU	roadside unit
OBU	onboard unit
TRA	a trace authority
$\left(T_{pub},t\right)$	TRA’s public key and private key
$RID_{i}$	$V_{i}$’s real identity
$PID_{i}$	$V_{i}$’s pseudo identity
$PK_{i}$	$V_{i}$’s public key
$R_{i},d_{i}$	$V_{i}$’s partial private key
$x_{i}$	$V_{i}$’s secret value
$T_{i}$	the valid period of $PID_{i}$
⊕	OR operation
$ct_{i}$	current timestamp
$M_{i}$	a message sent from vehicle to RSU
$P_{i}$	$V_{i}$’s public key in [12,13,14]
$\left(R_{i},S_{i}\right)$	a signature on $M_{i}$ in [12,13]
$\left(U_{i},V_{ijk}\right)$	a signature on $M_{i}$ in [14]

**Table 2:** Security comparisons.

Security	[12]	[13]	[14]	[15]	The Proposed Scheme
Authentication and Message integrity	✗	✓	✗	✓	✓
Identity privacy preserving	✓	✓	✓	✓	✓
Traceability	✓	✓	✓	✓	✓
Unlinkability	✓	✓	✓	✓	✓
Role separation	✓	✓	✗	✗	✓
Key escrow resilience	✓	✓	✗	✓	✓
Resistance to attacks	✗	✓	✗	✓	✓

**Table 3:** Execution time of cryptographic operation (in Milliseconds).

Cryptographic Operation	Execution Time
Bilinear pairing $T_{p}$	9.0791
Scalar multiplication in bilinear pairing $T_{m-bp}$	3.7770
Scalar multiplication in ECC $T_{m-ecc}$	0.8310
Map-to-point hash function in bilinear pairing $T_{mtp}$	9.7052

**Table 4:** Comparison of computation cost.

Scheme	A Message Signing	A Message Verification	*n* Message Signing	*n* Message Verification (Batch)
Hong et al’s scheme [12]	7.5540 ms	40.7195 ms	7.5540*n* ms	13.4822*n* + 27.2373 ms
Li et al’s scheme [13]	17.2592 ms	50.4247 ms	17.2592*n* ms	13.4822*n* + 36.9425 ms
Malhi et al’s scheme [14]	15.1080 ms	38.5683 ms	15.1080*n* ms	11.3310*n* + 27.2373 ms
The proposed scheme	0.8310 ms	3.3240 ms	0.8310*n* ms	3.3240*n* ms

**Table 5:** Comparison of communication cost.

Scheme	Send a Message	Send *n* Messages
Horng er al.’s scheme [12]	351 bytes	351*n* bytes
Li et al.’s scheme [13]	351 bytes	351*n* bytes
Malhi et al.’s scheme [14]	323 bytes	323*n* bytes
**The proposed scheme**	**128 bytes**	**128*n* bytes**

**Table 6:** Simulation parameters.

Parameters	Values
Simulation area	1000 m×1000 m
Wireless protocol	802.11 p
Channel bit rate	6 Mbs
Buffer size	1 M bytes
Number of RSU	9
Simulation time	200s
Traffic simulation tool	SUMO
Network simulation tool	ns-3.26
Vehicle speed	10–50 m/s

## Appendix A.

**Proof** **of** **Lemma** **1.** Assuming that a Type I adversary $A_{1}$ can break the proposed CLS-MR in time *t* with probability ε, there exists an algorithm *B* that can solve ECDL problem by utilizing $A_{1}$ as subroutine. Given a random instance {P,xP=Q} of the ECDL problem, the task of *B* is to compute *x*. Setup: The algorithm *B* sets $P_{pub}=Q$ and sends system parameters params to $A_{1}$. Here, hash functions $H_{1},H_{2},H_{3}$ are considered as random oracles in the proof. To keep the consistency and rapidly response, *B* maintains the initially empty lists as follows:

- $H_{1}$ list $L_{H_{1}}^{list}$: This list consists of tuples $\left(ID_{i},R_{i},c_{i}\right)$.
- $H_{2}$ list $L_{H_{2}}^{list}$: This list consists of tuples $\left(ID_{i},P_{pub},P_{i},l_{i}\right)$.
- $H_{3}$ list $L_{H_{3}}^{list}$: This list consists of tuples $\left(ID_{i},P_{pub},R_{i},u_{i},h_{i}\right)$.
- $L_{PPK}^{list}$: This list consists of tuples $\left(ID_{i},R_{i},d_{i}\right)$.
- $L_{SK}^{list}$: This list consists of tuples $\left(ID_{i},P_{i},x_{i}\right)$.

$H_{1}$ queries: Suppose $A_{1}$ submits a query on $\left(ID_{i},R_{i}\right)$, *B* checks $L_{H_{1}}^{list}$ and executes as follows:

- If the list $L_{H_{1}}^{list}$ includes $\left(ID_{i},R_{i},c_{i}\right)$, *B* responds with previous value $c_{i}=H_{1}\left(ID_{i},R_{i}\right)$ to $A_{1}$.
- If the list $L_{H_{1}}^{list}$ does not include $\left(ID_{i},R_{i},c_{i}\right)$, *B* randomly chooses $c_{i}\in Z_{q}$ , adds $\left(ID_{i},R_{i},c_{i}\right)$ in $L_{H_{1}}^{list}$ and sends $c_{i}=H_{1}\left(ID_{i},R_{i}\right)$ to $A_{1}$.

$H_{2}$ queries: Suppose $A_{1}$ submits a query on $\left(ID_{i},P_{pub},P_{i}\right)$, *B* checks $L_{H_{2}}^{list}$ and executes as follows:

- If the list $L_{H_{2}}^{list}$ includes $\left(ID_{i},P_{pub},P_{i},l_{i}\right)$, *B* responds with previous value $l_{i}=H_{2}\left(ID_{i},P_{pub},P_{i}\right)$ to $A_{1}$.
- If the list $L_{H_{2}}^{list}$ does not include $\left(ID_{i},P_{pub},P_{i},l_{i}\right)$, *B* randomly chooses $l_{i}\in Z_{q}$, adds $\left(ID_{i},P_{pub},P_{i},l_{i}\right)$ in $L_{H_{2}}^{list}$ and sends $l_{i}=H_{2}\left(ID_{i},P_{pub},P_{i}\right)$ to $A_{1}$.

$H_{3}$ queries: Suppose $A_{1}$ submits a query on $\left(ID_{i},P_{pub},R_{i},u_{i}\right)$, *B* checks $L_{H_{3}}^{list}$ and executes as follows:

- If the list $L_{H_{3}}^{list}$ includes $\left(ID_{i},P_{pub},R_{i},u_{i},h_{i}\right)$, *B* responds with previous value $h_{i}=H_{3}\left(ID_{i},P_{pub},R_{i},u_{i}\right)$ to $A_{1}$.
- If the list $L_{H_{3}}^{list}$ does not include $\left(ID_{i},P_{pub},R_{i},u_{i},h_{i}\right)$, *B* randomly chooses $h_{i}\in Z_{q}$, adds $\left(ID_{i},P_{pub},R_{i},u_{i},h_{i}\right)$ in $L_{H_{3}}^{list}$ and sends $h_{i}=H_{3}\left(ID_{i},P_{pub},R_{i},u_{i}\right)$ to $A_{1}$.

Partial private key queries: Suppose $A_{1}$ submits a partial private key query on the identity $ID_{i}$, *B* checks $L_{PPK}^{list}$ and executes as follows:

- If the list $L_{PPK}^{list}$ includes $\left(ID_{i},R_{i},d_{i}\right)$, *B* responds with previous value $\left(R_{i},d_{i}\right)$ to $A_{1}$.
- If the list $L_{PPK}^{list}$ does not include $\left(ID_{i},R_{i},d_{i}\right)$, *B* picks random numbers $d_{i},c_{i}\in Z_{q}$ and sets $c_{i}=H_{1}\left(ID_{i},R_{i}\right)$ and $R_{i}=d_{i}P-c_{i}P_{pub}$. Finally, *B* outputs the $\left(R_{i},d_{i}\right)$ to $A_{1}$, and inserts the $\left(ID_{i},R_{i},c_{i}\right)$ and $\left(ID_{i},R_{i},d_{i}\right)$ to $L_{H_{1}}^{list}$ and $L_{PPK}^{list}$, respectively.

Secret value queries: Suppose $A_{1}$ submits a secret value query on the identity $ID_{i}$, *B* checks $L_{SK}^{list}$ and executes as follows:

- If the list $L_{SK}^{list}$ includes $\left(ID_{i},P_{i},x_{i}\right)$, *B* responds with previous value $x_{i}$ to $A_{1}$.
- If the list $L_{SK}^{list}$ does not include $\left(ID_{i},P_{i},x_{i}\right)$, *B* randomly chooses $x_{i}\in Z_{q}^{*}$ and computes $P_{i}=x_{i}P$. Finally, *B* returns $x_{i}$ to $A_{1}$, and inserts the $\left(ID_{i},P_{i},x_{i}\right)$ to $L_{SK}^{list}$.

Public key queries: Suppose $A_{1}$ submits a public key query on the identity $ID_{i}$, *B* checks $L_{PPK}^{list}$, $L_{SK}^{list}$ and executes as follows:

- If the list $L_{PPK}^{list}$ includes $\left(ID_{i},R_{i},d_{i}\right)$ and the list $L_{SK}^{list}$ includes $\left(ID_{i},P_{i},x_{i}\right)$, *B* responds with previous value $\left(R_{i},P_{i}\right)$ to $A_{1}$.
- If the list $L_{PPK}^{list}$ does not include $\left(ID_{i},R_{i},d_{i}\right)$ or $L_{SK}^{list}$ does not include $\left(ID_{i},P_{i},x_{i}\right)$, *B* issues a partial private key query or secret value query itself on $ID_{i}$. Finally, *B* returns $\left(R_{i},P_{i}\right)$ to $A_{1}$, and inserts the corresponding values to $L_{PPK}^{list}$ and $L_{SK}^{list}$.

Public key replacement queries: Suppose $A_{1}$ submits a public key replacement query on $\left\{ID_{i},R_{i}^{\prime }\;,P_{i}^{\prime }\;\;\right\}$, *B* checks $L_{PPK}^{list}$, $L_{SK}^{list}$ and executes as follows:

- If the list $L_{PPK}^{list}$ includes $\left(ID_{i},R_{i},d_{i}\right)$ and the list $L_{SK}^{list}$ includes $\left(ID_{i},P_{i},x_{i}\right)$, *B* sets $R_{i}=R_{i}^{\prime }$, $P_{i}=P_{i}^{\prime }$, $d_{i}=⊥$, $x_{i}=⊥$ and updates $\left(ID_{i},R_{i},d_{i}\right)$, $\left(ID_{i},P_{i},x_{i}\right)$ to the list $L_{PPK}^{list}$ and $L_{SK}^{list}$, respectively.
- If the list $L_{PPK}^{list}$ does not include $\left(ID_{i},R_{i},d_{i}\right)$ or the list $L_{SK}^{list}$ does not include $\left(ID_{i},P_{i},x_{i}\right)$, *B* sets $R_{i}=R_{i}^{\prime }$, $P_{i}^{\prime }=P_{i}^{\prime }$, $d_{i}=⊥$, $x_{i}=⊥$ and inserts $\left(ID_{i},R_{i},d_{i}\right)$, $\left(ID_{i},P_{i},x_{i}\right)$ to the list $L_{PPK}^{list}$ and $L_{SK}^{list}$, respectively.

Sign queries: Suppose $A_{1}$ submits a sign query on $\left(m,ID_{i},R_{i},P_{i}\right)$, *B* firstly conducts a partial private key query itself to generate $\left(R_{i},d_{i}\right)$. *B* randomly chooses $v_{i}\in Z_{q}^{*}$ and computes $f=F_{1}\left(m\right)\left|\right|F_{2}\left(F_{1}\left(m\right)\right)⊕m$, $u_{i}=f⊕\left(v_{i}P-l_{i}P_{i}-h_{i}R_{i}-h_{i}c_{i}P_{pub}\right)$. If the tuple including $h_{i}$ already appears on $L_{H_{3}}^{list}$, *B* selects another $v_{i}\in Z_{q}^{*}$ and tries again. Finally, *B* returns $\left\{u_{i},v_{i}\right\}$ to $A_{1}$. Forgery: $A_{1}$ outputs a valid signature $\left(u_{i}^{*},v_{i}^{*}\right)$ on $m^{*}$ under $\left(ID_{i}^{*},R_{i}^{*},P_{i}^{*}\right)$. Using the Forking Lemma [48], *B* can obtain another valid signature $\left(u_{i}^{*},{v_{i}^{*}}^{\prime }\right)$ under $\left(ID_{i}^{*},R_{i}^{*},P_{i}^{*}\right)$ by replaying the process with the same random tape, yet with a different choice of $H_{1}$. Then, we have

$$v_{i}^{*}P-l_{i}^{*}P_{i}-h_{i}^{*}R_{i}-h_{i}^{*}c_{i}^{*}P_{pub}={v_{i}^{*}}^{\prime }P-l_{i}^{*}P_{i}-h_{i}^{*}R_{i}-h_{i}^{*}{c_{i}^{*}}^{\prime }P_{pub},$$

$$v_{i}^{*}P-h_{i}^{*}c_{i}^{*}P_{pub}=v_{i}^{*}P_{i}-h_{i}^{*}c_{i}^{*}P_{pub}.$$

From the above equation, we obtain

$$\left(v_{i}^{*}-{v_{i}^{*}}^{\prime }\right)P=\left(h_{i}^{*}c_{i}^{*}-h_{i}^{*}{c_{i}^{*}}^{\prime }\right)xP$$

. Finally, *B* outputs the solution to ECDL problem $x=h_{i}^{*-1}{\left(c_{i}^{*}-c_{i}^{{*}^{\prime }}\right)}^{-1}\left(v_{i}^{*}-v_{i}^{{*}^{\prime }}\right)$. After completing the above simulation, we will analyze the *B*’s probability and time for solving the ECDL problem. Let us assume that $A_{1}$ can make at most $q_{H_{i}}$$H_{i}\left(i=1,2,3\right)$ queries, $q_{pp}$ partial private key queries, $q_{sv}$ secret value queries, $q_{pk}$ public key queries, $q_{pr}$ public key replacement queries, and $q_{s}$ times sign queries. The probability of failure in making a partial private key query caused by a conflict on is $H_{1}$ most $\frac{q_{H_{1}}q_{pp}}{q}$. The probability of failure in issuing a sign query resulting from a conflict on $H_{3}$ is at most $\frac{q_{s}\left(q_{H_{3}}+q_{s}\right)}{q}$. In addition, the probability of $A_{1}$ outputs a valid forgery without asking the corresponding $H_{1},H_{2},H_{3}$ is at most $\frac{3}{q}$. The probability of *B* correctly guesses it as the point of rewind is at least $\frac{1}{q_{H_{1}}}$. Therefore, the success probability of *B* for solving the ECDL problem is at least ε-(qH1qpp+qs(qH3+qs)+3)(qH1qpp+qs(qH3+qs)+3)qqqH1. The running time of *B* is equal to the running time of $A_{1}$ plus the time it takes to respond to $q_{pp}$ partial private key queries, $q_{sv}$ secret value queries, $q_{pk}$ public key queries and $q_{s}$ sign queries. Each partial private key query requires 2 scale multiplication operations in 𝔾. Each secret value query requires 1 scale multiplication operation in 𝔾. Each public key query requires 1 scale multiplication operation in 𝔾. Each sign query requires 4 scale multiplication operations in 𝔾. Assuming that each scale multiplication in 𝔾 needs time $t_{sm}$, the total running time of *B* is at most $t+\left(2q_{pp}+q_{sv}+q_{pk}+4q_{s}\right)t_{sm}$. ☐

## Appendix B.

**Proof** **of** **Lemma** **2.** Assuming that a Type II adversary $A_{2}$ can break the proposed CLS-MR in time *t* with probability ε, there exists an algorithm *B* that can solve ECDL problem by utilizing $A_{2}$ as subroutine. Given a random instance {P,xP=Q} of the ECDL problem, the task of *B* is to compute *x*. Setup: The algorithm *B* randomly selects $\theta \in Z_{q}$ and defines $\theta P=P_{pub}$; then, *B* sends the system parameters params and master key θ to $A_{2}$. Note that $A_{2}$ has the master key and does not require to issue any partial private key query. Similar to Lemma 1, the lists $L_{H_{1}}^{list}$, $L_{H_{2}}^{list}$, $L_{H_{3}}^{list}$, $L_{PPK}^{list}$ and $L_{SK}^{list}$ are maintained by *B*. *B* also keeps a list $L_{}^{list}=\left(ID_{i},P_{i},x_{i},z_{i}\right)$, which is initial-empty. $H_{1}$, $H_{2}$ and $H_{3}$ queries: It is the same as Lemma 1. Secret value queries: Suppose $A_{2}$ submits a secret value query on the identity $ID_{i}$, *B* checks $L_{}^{list}$ and executes as follows:

- If the list $L_{}^{list}$ includes $\left(ID_{i},P_{i},x_{i},z_{i}\right)$, if $z_{i}=0$, *B* halts; if $z_{i}=1$, *B* responds with previous value $x_{i}$ to $A_{2}$.
- If the list $L_{}^{list}$ does not include $\left(ID_{i},P_{i},x_{i},z_{i}\right)$, using the Coron’s technique [49], *B* tosses a coin $z_{i}\in \left\{0,1\right\}$ that produces 0 with probability δ and 1 with probability 1-δ. *B* randomly chooses a value $x_{i}\in Z_{q}$. If $z_{i}=0$, *B* sets $P_{i}=x_{i}Q$ ; if $z_{i}=1$, *B* sets $P_{i}=x_{i}P$. Finally, *B* inserts the $\left(ID_{i},P_{i},x_{i},z_{i}\right)$ to $L_{}^{list}$. If $z_{i}=0$, *B* halts; if $z_{i}=1$, *B* responds the value $x_{i}$ to $A_{2}$.

Public key queries: Suppose $A_{2}$ submits a public key query on the identity $ID_{i}$, *B* checks $L_{}^{list}$ and executes as follows:

- If the list $L_{}^{list}$ includes $\left(ID_{i},P_{i},x_{i},z_{i}\right)$, *B* responds with previous value $P_{i}$ to $A_{2}$.
- If the list $L_{}^{list}$ does not include $\left(ID_{i},P_{i},x_{i},z_{i}\right)$, *B* submits a secret value query on $ID_{i}$ and returns $P_{i}$ to $A_{2}$ . Here, $A_{2}$ can obtain $R_{i}$ corresponding to $D_{i}$ using the master key.

Sign queries: It is the same as Lemma 1. Forgery: $A_{2}$ outputs a valid signature $\left(u_{i}^{*},v_{i}^{*}\right)$ on $m^{*}$ under $\left(ID_{i}^{*},R_{i}^{*},P_{i}^{*}\right)$. Using the Forking Lemma [48], *B* can obtain another valid signature $\left(u_{i}^{*},{v_{i}^{*}}^{\prime }\right)$ on $m^{*}$ under $\left(ID_{i}^{*},R_{i}^{*},P_{i}^{*}\right)$ by replaying process under the same random tape with a different choice of $H_{2}$. Then, we have

$$v_{i}^{*}P-l_{i}^{*}P_{i}-h_{i}^{*}R_{i}-h_{i}^{*}c_{i}^{*}P_{pub}={v_{i}^{*}}^{\prime }P-{l_{i}^{*}}^{\prime }P_{i}-h_{i}^{*}R_{i}-h_{i}^{*}c_{i}^{*}P_{pub},$$

$$v_{i}^{*}P-l_{i}^{*}P_{i}={v_{i}^{*}}^{\prime }P_{i}-{l_{i}^{*}}^{\prime }P_{i}.$$

From the above equation, *B* checks the $L_{}^{list}$, if $c_{i}^{*}=1$, *B* aborts; if $c_{i}^{*}=0$, the above equation, we have

$$\left(v_{i}^{*}-{v_{i}^{*}}^{\prime }\right)P=\left(l_{i}^{*}-{l_{i}^{*}}^{\prime }\right)x_{i}xP$$

. Finally, *B* outputs *x* by computing $x=x_{i}^{{*}^{-1}}{\left(l_{i}^{*}-l_{i}^{{*}^{\prime }}\right)}^{-1}\left(v_{i}^{*}-v_{i}^{{*}^{\prime }}\right)$, which is the solution to the ECDL problem. The same as Lemma 1, the analysis on the probability and time of *B* is as follows, assuming that $A_{2}$ can make at most $q_{H_{i}}$$H_{i}\left(i=1,2,3\right)$ queries, $q_{sv}$ secret value queries, $q_{pk}$ public key queries, and $q_{s}$ sign queries. The probability of failure in handing a sign query because of a conflict on $q_{H_{3}}$ is at most $\frac{q_{s}\left(q_{H_{3}}+q_{s}\right)}{q}$. In a secret value query and forgery phase, the probability of success is ${\left(1-\delta \right)}^{q_{sv}}\delta$ according to Coron’s technique [49]. When the optimal probability is $\delta =\frac{1}{q_{sv}+1}$, it is greater than $\frac{1}{e(q_{sv}+1)}$. The probability of $A_{2}$ outputs a valid forgery signature without asking the corresponding $H_{1}$ or $H_{2}$ or $H_{3}$ is at most $\frac{3}{q}$. The probability of *B* correctly guesses it, as the point of rewind is at least $\frac{1}{q_{H_{2}}}$. Therefore, the success probability of *B* for solving the ECDL problem is at least ε-(qs(qH3+qs)+3)(qs(qH3+qs)+3)qqe(qsv+1)qH2. The running time of *B* is equal to the running time of $A_{2}$ plus the time it takes to respond to $q_{sv}$ secret value queries, $q_{pk}$ public key queries and $q_{s}$ sign queries. Each secret value query requires one scale multiplication operation in 𝔾. Each public key query requires one scale multiplication operation in 𝔾. Each sign query requires four scale multiplication operations in 𝔾. Assuming that each scale multiplication in 𝔾 needs time $t_{sm}$, the total running time of *B* is at most $t+\left(q_{cv}+q_{pk}+4q_{s}\right)t_{sm}$. ☐
